# A Volatile and Dynamic Longitudinal Microbiome Is Associated With Less Reduction in Lung Function in Adolescents With Cystic Fibrosis

**DOI:** 10.3389/fcimb.2021.763121

**Published:** 2021-12-06

**Authors:** Marisa I. Metzger, Simon Y. Graeber, Mirjam Stahl, Olaf Sommerburg, Marcus A. Mall, Alexander H. Dalpke, Sébastien Boutin

**Affiliations:** ^1^ Department of Infectious Diseases, Medical Microbiology and Hygiene, University of Heidelberg, Heidelberg, Germany; ^2^ Translational Lung Research Center Heidelberg (TLRC), German Center for Lung Research (DZL), University of Heidelberg, Heidelberg, Germany; ^3^ German Centre for Lung Research (DZL), Associated Partner Site, Berlin, Germany; ^4^ Department of Pediatric Respiratory Medicine, Immunology and Critical Care Medicine and Cystic Fibrosis Center, Charité - Universitätsmedizin Berlin, Berlin, Germany; ^5^ Berlin Institute of Health (BIH), Berlin, Germany; ^6^ Division of Pediatric Pulmonology & Allergy and Cystic Fibrosis Center, Department of Pediatrics, University of Heidelberg, Heidelberg, Germany; ^7^ Department of Translational Pulmonology, University of Heidelberg, Heidelberg, Germany; ^8^ Institute of Medical Microbiology and Virology, Technische Universität Dresden, Dresden, Germany

**Keywords:** lung microbiome, cystic fibrosis, longitudinal study, volatility analysis, *Pseudomonas aeruginosa*, adolescent

## Abstract

Progressive impairment in lung function caused by chronic polymicrobial airway infection remains the major cause of death in patients with cystic fibrosis (CF). Cross-sectional studies suggest an association between lung function decline and specific lung microbiome ecotypes. However, longitudinal studies on the stability of the airway microbiome are missing for adolescents with CF constituting the age group showing the highest rate of decline in lung function. In this study, we analyzed longitudinal lung function data and sputum samples collected over a period of 3 to 5 years from 12 adolescents with CF. The sputum microbiome was analyzed using 16S rRNA gene sequencing. Our results indicate that the individual course of the lung microbiome is associated with longitudinal lung function. In our cohort, patients with a dynamic, diverse microbiome showed a slower decline of lung function measured by FEV_1%_ predicted, whereas a more stable and less diverse lung microbiome was related to worse outcomes. Specifically, a higher abundance of the phyla Bacteroidetes and Firmicutes was linked to a better clinical outcome, while Proteobacteria were correlated with a decline in FEV_1%_ predicted. Our study indicates that the stability and diversity of the lung microbiome and the abundance of Bacteroidetes and Firmicutes are associated with the lung function decline and are one of the contributing factors to the disease severity.

## Introduction

Patients with the autosomal recessive disorder cystic fibrosis (CF) suffer progressive impairment of lung function, which is the most common reason for reduced quality of life and mortality (Bell et al., 2020; Mall et al., 2020). Infections with potential respiratory pathogens (PRP) increase mortality (Sherrard et al., 2014). PRP such as *Pseudomonas aeruginosa* or *Staphylococcus aureus* may overgrow the commensal microbiota of the airways causing a decreased microbial diversity in the lung, which is associated with a decrease in lung function and an increase of pulmonary exacerbations in adult CF patients (Boutin et al., 2015; Coburn et al., 2015; O’Neill et al., 2015; Boutin et al., 2017).

Analysis of the lung microbiome in cross-sectional studies revealed variable microbial compositions among patients with CF (Coburn et al., 2015). Of note, the lung microbiome of pediatric patients with CF was shown to be more diverse as compared to adult patients with more advanced lung disease states (Cox et al., 2010; Zemanick et al., 2017). Furthermore, it was demonstrated that patients harbor an individual lung microbiome; thus, longitudinal instead of cross-sectional studies are required to reveal the functional role of microbiome alterations (Whelan et al., 2017).

A few longitudinal studies in CF patients have already been performed (Carmody et al., 2015; Cuthbertson et al., 2016; Whelan et al., 2017; Ahmed et al., 2019) confirming a patient-specific microbiome. No overall community change in the microbiome could be detected upon the first exacerbation in infants (Ahmed et al., 2019), as well as in adults with CF (Whelan et al., 2017). The PRP *P. aeruginosa* was reported to occur in a high relative amount during an exacerbation but also in phases of stable lung function. Cuthbertson et al. reported no change in the microbiome community upon exacerbation and antibiotic treatment indicating a high resistance and resilience of the lung microbiome (Cuthbertson et al., 2016).

We focused on the adolescent age group when the microbiome often shifts from a diverse microbiome to a stable microbiome with a higher load of PRPs, which goes along with a fast decline in lung function and an increase of the CF disease’s burden (Szczesniak et al., 2017). The aim of our study was to evaluate the long-term evolution of the microbiome over a period of more than 3 years. We hypothesized that the personalized lung microbiome of each patient is related to lung disease progression and that patients with a better lung function show a more diverse, volatile lung microbiome.

## Material and Methods

### Study Subjects and Design

Adolescent patients, diagnosed with CF based on established diagnostic criteria (De Boeck et al., 2011), provided sputum samples *via* spontaneous expectorations during clinical visits. These included routine visits every 3 months and unforeseen visits due to pulmonary exacerbations and elective treatments. The study was approved by the Ethics Committee of the University of Heidelberg (S-370/2011), and all patients, their parents, and/or legal guardians gave written informed consent. Only patients having expectorated sputum at least during three routine visits per year during the full course of 3 to 5 years were included, resulting in 12 patients included in the study. Clinical data including demographics, lung function, and information on antibiotic therapy are provided in **Table 1**. Lung function was determined by the forced expiratory volume in one second expressed as percent predicted (FEV_1_%pred) according to the global lung function initiative reference values (Cooper et al., 2017). Infections with *P. aeruginosa* were classified as negative, intermittent, or chronic based on culture results and *P. aeruginosa* antibody titers as previously described (Stahl et al., 2017). None of the patients were under CFTR modulator therapy during the course of the study.

**Table 1:** Clinical characteristics of patients with cystic fibrosis.

	Stable	Decliner
Number of subjects	4	8
Age in years (sd) NS	17.1 (1.18)	17.3 (1.4)
Sex males/females	2/2	3/5
BMI [kg/m^2^] (sd)***	20.5 (1.41)	18.4 (2.04)
FEV_1%_ predicted (sd)***	86.8 (16.3)	58.2 (22.5)
CFTR genotype		
F508del/F508del	1	3
F508del/other	3	4
other/other		1
Number if i.v. treatment per year (sd)**	1.1 (0.5)	3.5 (1.77)
Pancreatic insufficiency	4	8

Data are expressed as mean with the standard deviation. The difference in age, BMI, and FEV_1%_ predicted between the two groups was evaluated by a t-test. **p < 0.05), ***p < 0.001, NS, not significant.

BMI, body mass index, FEV_1_, forced expiratory volume in 1 s.

### Sputum Microbiome Analysis

Details are provided in the online supplement. After sample preparation, DNA was extracted with the QIAamp Mini Kit (Qiagen, Hilden, Germany) according to the manufacturer’s protocol. DNA amplification *via* PCR was done using universal barcoded bacterial primers flanking the V4 region of the 16S rRNA gene (515F: GTGCCAGCMGCCGCGGTAA and 806R: GGACTACHVGGGTWTCTAAT). A mock community (HM-782D, BEI Resources, Manassas, USA) was used in the PCR as a positive control. Next-generation paired-end sequencing was performed on an Illumina MiSeq system (2 × 250 cycles) by Eurofins Genomics. Processing the raw sequences was done using DADA2 as previously described to amplicon sequence variants (ASV) (Schmitt et al., 2020). Shortly, raw sequences were processed through quality control (no ambiguities (N), less than one error per reads, truncation of the reads after quality score < 2) merged as contigs and checked for chimera with the default parameters of DADA2. Taxonomy was defined based on the Silva database (Version 132). Negative control from DNA extraction and PCR reaction were performed to evaluate contamination. However, none of the blanks produced a visible amplicon (QIAxcel DNA screen) or showed enough DNA to be quantified with PicoGreen (<2.5 ng/ml), indicating no contamination. A quantitative PCR was performed with a universal 16S rRNA primer to evaluate the number of 16S rRNA gene copies as previously published (Boutin et al., 2017; Schoilew et al., 2019).

### Statistics

A time index (t_i_) was created starting at the 14th year for each patient to align all patients accordingly to their age. The time range of this study is monthly to obtain rational numbers needed for the volatility analysis. Descriptive indices and statistics were calculated using the R packages *phyloseq* (McMurdie and Holmes, 2013) and *microbiome* (Leo Lahti et al., 2017). Statistical variation of the β-diversity and clinical values was investigated by permutational analysis of variance (PERMANOVA) with the *vegan* R package (Jari Oksanen et al., 2019). Variation of descriptive values and clinical values between groups was tested with a Wilcoxon rank-sum test. The R packages *stats* (Team RC, 2019) and *cluster* (Maechler et al., 2019) were used to perform hierarchical cluster analysis on the samples. The hierarchical cluster tree based on *Ward’s minimum variance* was used to calculate the cluster groups of all microbiome samples. A phylogenetic tree of all ASVs was constructed by multiple-sequence alignment with the *DECIPHER* R package (Wright, 2016), and the constructed neighbor-joining tree served as a template for a maximum likelihood tree calculated by the *phangorn* R package (Schliep, 2011). The *longitudinal* plugin for Qiime2 (Bokulich et al., 2018) was used to perform metadata Volatility Analysis and Feature Volatility Analysis at the ASV level and phylum level. In the “feature volatility analysis,” the machine learning algorithm uses the structure of the data as a learning input to identify features (ASV or phyla) that are the most important ones in the prediction of the different states (= time points). The n_estimators input value was set to 1,000, and Random Forest Regression was used as an estimator. The details are provided in the online supplement. Raw data and scripts are available in the GitHub repository https://github.com/MarisaIsabell/CF-lung-microbiome-longitudinal-development. All analysis in R was done with R 3.6.0 and RStudio 1.1.463.

## Results

### Patient With a Declining Lung Function Showed a Higher Number of Intravenous Therapies per Year

We were able to collect 286 sputum samples from 12 patients with at least three samples per year over a 3–5-year period (**Supplementary Figure 1**). To group patients for different functional outcomes, we calculated the change of the mean FEV_1_%pred per year with a linear regression and used the slope as the rate of decline per year. By using a grouping threshold of 0%, we defined “stable” patients (n = 4) showing a stable FEV_1_%pred over the years with the change ranging from 0.34% to 1.84% per year. The “decliner” patients (n = 8) had a decline of the FEV_1_%pred values between -0.08% and -9.14% per year.

We then analyzed this dynamic, functional parameter in comparison to different demographic and clinical variables. We observed that the number of intravenous (i.v.) antibiotic therapies per year correlated with the change of FEV_1_%pred per year: Patients showing a decline in lung function experienced more i.v. antibiotic therapies (**Supplementary Figure 1**). Antibiotic therapy is a known to have a high influence of the microbiome. Therefore, we compared the impact of the number of i.v. antibiotic therapies per year on the median values of each patient’s microbiome alpha diversity (Shannon index), dominance (relative), and evenness (Simpson index) and on the microbial burden (copies/µl). We observed no significant relationship between the numbers of i.v. antibiotic therapies per year on either alpha-diversity parameter.

### The Temporal Evolution of the Lung Microbiome Is Driven by a Personalized Microbial Fingerprint

When analyzing the airway microbiome, we identified the individual patient as a factor in the PERMANOVA explaining 49% (Morisita-Horn distance) or 33% (weighted UniFrac distance) of the variance. The β-diversity calculated between two samples from the same patient at different time points was significantly lower (p < 0.001) than the β-diversity between two samples from different patients. Thus, the microbiomes of individual patients in this cohort stayed more similar over time compared to the microbiome of different patients. These results show that each patient harbors a dynamic but individual microbiome evolution.

### Patients With Stable Lung Function Have a More Diverse Microbiome With Less Dominance

**Figure 1:**
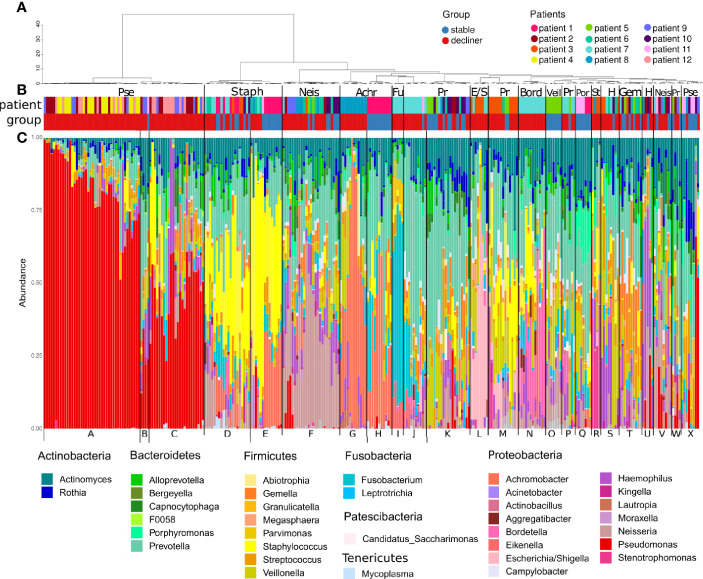
Microbiome composition of each sample grouped by hierarchical clustering. **(A)** Dendrogram representing the hierarchical clustering of the samples. **(B)** Colored bars showing the affiliation of the samples to a patient (pat) and the patient FEV-group (group; stable or decliner). The most abundant genera are written above the colored bars to name the clusters. Pse, *Pseudomonas*; Staph, *Staphylococcus*; Neis, *Neisseria*; Achr, *Achromobacter*; Fu, *Fusobacteria*; Pr, *Prevotella*; E/S, *Escherichia/Shigella*; Bord, *Bordetella*; Veil, *Veillonella*; Por, *Porphyromonas*; St, *Stenotrophomonas*; H, *Haemophilus*; Gem, *Gemella.* **(C)** Relative abundance of the genera, which occurs in at least one sample with a relative abundance ≥5%. All other genera are cumulated as “Others.” The letter at the bottom indicates the cluster groups.

We found 137 distinct ASVs and 38 genera, which were present in at least one sample with a relative abundance ≥5%. All samples were clustered based on their microbiome composition in 24 hierarchical clusters (**Figure 1**). Cluster A consists mostly of samples with high abundance of *Pseudomonas* (relative abundance > 60%) and was characteristic for samples from patients with a chronic infection of the mentioned bacteria. Related clusters were cluster C with a mid-level *Pseudomonas* infection (relative abundance primary > 25%) and cluster B with samples harboring a low-level infection (relative abundance primary < 25%). Indeed, the clinical data reported chronic infection with *Pseudomonas aeruginosa* (**Supplementary Figure 1**) for patients 2, 4, 9, and 12, whose samples mostly build up these clusters.

The genus *Pseudomonas* was almost exclusively present in the “decliner” population, suggesting a worsening of the lung function associated with the infection with the pathogen (**Figure 1B**). The β-diversity from samples from the same patient affiliated to the *Pseudomonas* clusters was much lower than the β-diversity within the commensal clusters (**Supplementary Figure 2**), indicating a less diverse and more stable microbiome for *Pseudomonas*-infected samples. Other reported pathogens in CF like *Staphylococcus* and *Haemophilus* dominated clusters, which are composed of samples from both decliner and stable patients (cluster M, D, and S). Clusters dominated by commensals like *Porphyromonas* or *Veillonella* were composed of samples from stable patients.

**Figure 2:**
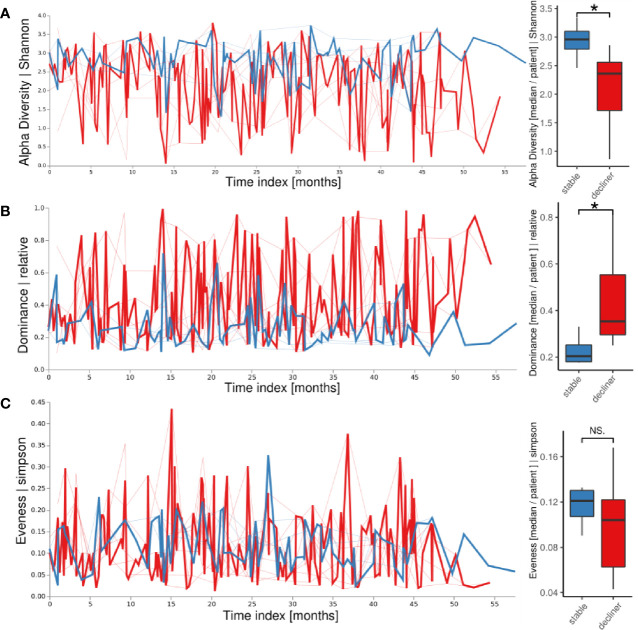
Longitudinal volatility analysis and boxplot visualization of descriptive microbiome statistics grouped by the patient groups: Alpha diversity **(A)**, relative dominance **(B)**, and the evenness **(C)**. The thick lines represent how the variable changes over time in the patient group while the thin lines represent each patient. *p < 0.05, NS, not significant.

We next intended to probe whether differences in individual fluctuations of the microbiome instead of the analysis of a static assignment into a microbial cluster would be more appropriate to link microbiome to clinical changes in individual patients. We used a volatility analysis in which the temporal development of each patient’s microbiome parameters (alpha diversity, dominance, evenness) was used to visualize the fluctuation of the corresponding descriptive parameter in two patient groups (**Figure 2A**). The α-diversity was higher in patients from the “stable” groups compared to the “decliner” group over the whole time period. When calculating the mean of the alpha diversity for each patient, the trend stayed the same (**Figure 2A**, boxplot; p < 0.05). Accordingly, the relative dominance, as analyzed in a longitudinal manner, was higher for the patients with a decline in FEV_1_%pred than for the “stable” patients (**Figure 2B**) with a significant p-value of 0.03 when comparing the patient means for relative dominance. Thus, patients with a decline in lung function had a less diverse and more dominated microbiome. Furthermore, the volatility analysis also clearly revealed the high variation of single-point measurements as revealed by the high variation of the microbial parameters, most pronounced in the decliner group.

The evenness of the microbiome did not reveal a large difference between the two patient groups in the longitudinal volatility analysis as well as in the boxplot comparing the mean evenness for each patient with a p-value of 0.57 (**Figure 2C**). The microbiome of the “decliner” population was indeed dominated by mostly one pathogen.

### The “Decliner” Group Is Dominated by Pseudomonas ASV While the “Stable” Group Showed Higher Abundance of Commensal Bacteria

**Figure 3:**
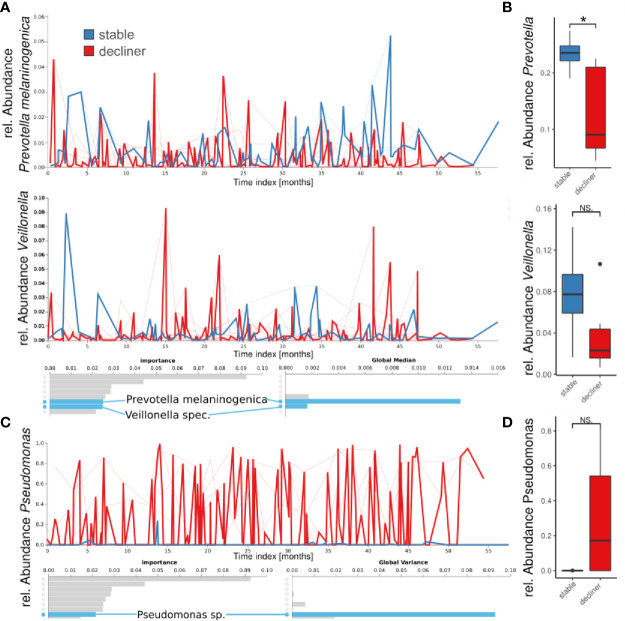
Longitudinal feature volatility analysis on ASV level **(A, C)** and boxplot visualization **(B, D)** grouped by patient groups. The feature volatility analysis was done on ASV levels, and the boxplots are median values for each patient for all species with the respective genus name. **(A, B)** Shown are two examples of important commensals (*Prevotella* and *Veillonella*), which do also reveal a high global median in the feature volatility analysis. **(C, D)** A *Pseudomonas* ASV is the eighth important feature (ASV), but the first with a very high global variance indicates a high impact on the microbiome development, and it is important for distinguishing between patient groups. *p < 0.05), NS, not significant.

We performed a “Feature Volatility Analysis” on the ASV level which can identify ASVs that are most discriminatory between the patient groups. The first seven ASVs reported by the Feature Volatility Analysis, which hold the highest importance for the random forest classifier to discriminate between the states, belonged to commensal bacteria (**Figure 3A**). Among those, *Prevotella melaninogenica* and *Veillonella* sp. had a high global median (referring to the relative abundance of the taxa in all samples) indicating the frequent presence in the samples. Together, high global median and identification by feature volatility analysis indicated the ubiquitous abundance of those commensals in the microbiome and importance for the ecology of the lung microbiome. Calculating the statistical difference between the relative abundances of the respective genera reported a decrease in the “decliner” group, which was significant for *Prevotella* (p = 0.03), but not for *Veillonella* (p = 0.15) (**Figure 3B**).

The first ASV with a very high global variance in the microbiome, indicating a great difference in relative abundance between the groups, was a *Pseudomonas* sp. The high variance and high global mean (not shown), but low global median, suggest that this ASV was not frequently found in the samples but dominated the microbiome if present. Indeed, the prevalence of *Pseudomonas* in our cohort (including all samples) was 54.2% with relative individual abundances up to almost 100% (**Figure 1C**). In the boxplot analysis based on the *Pseudomonas* mean abundance per patient, the decliner group revealed a higher relative abundance of the genus *Pseudomonas* (p = 0.3). We also observed a slightly higher bacterial burden in the sputum of patients from the decliner group (**Supplementary Figure 3**).

### The Phylum Proteobacteria Is the Main Driver for the “Decliner” Patient Group

**Figure 4:**
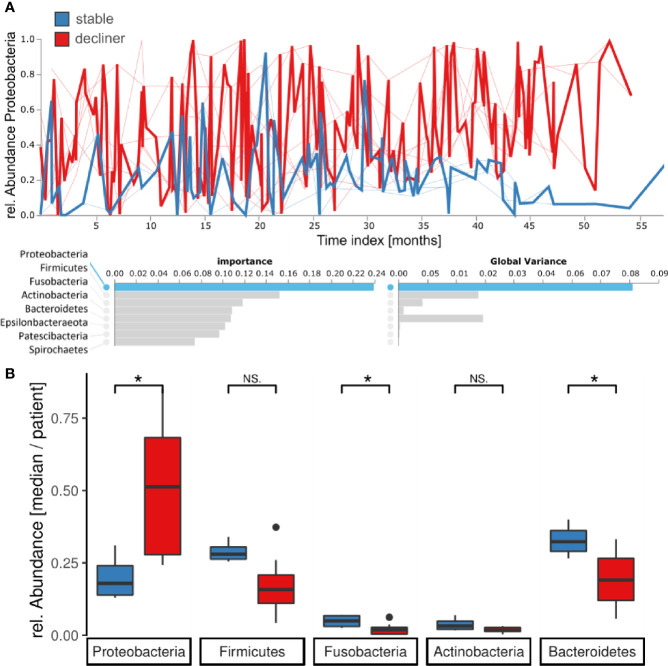
Proteobacteria are enriched for patients in the decliner group. **(A)** Longitudinal feature volatility analysis at the phylum level reveals the Proteobacteria as the most important feature for the microbiome progression in this study. **(B)** Median relative abundance for each patient of the five most important phyla grouped by the patient groups. *p < 0.05, NS, not significant.

At the phylum level, the Feature Volatility Analysis reported Proteobacteria as the most important phylum to distinguish the longitudinal microbiome development between the two patient groups, which also comprises *Pseudomonas* (**Figure 4A**). In the volatility plot, the “stable” patients had a lower amount of Proteobacteria in their microbiome, whereas the “decliner” patients harbored more Proteobacteria. For the Proteobacteria phylum volatility as well as the *Pseudomonas* volatility, the graph for the “decliner” group is more variable than for the “stable” group. We observed an enrichment of the Proteobacteria (p = 0.03) but a decrease of the next following important phyla Firmicutes (p = 0.07), Fusobacteria (p = 0.05), Actinobacteria (p = 0.15), and Bacteroides (p = 0.05) in the “decliner” compared to the “stable” patients (**Figure 4B**). The non-significance for the differences observed in Firmicutes, Fusobacteria, and Actinobacteria when comparing the mean per patient for those values is not surprising due to the low number of patients and thus data points for this comparison.

## Discussion

This study with 12 patients and the continuous sampling for up to 5 years is to our knowledge the first long-term longitudinal study in adolescents with CF. Other studies have focused on infants (Frayman et al., 2017; Ahmed et al., 2019) or adults (Whelan et al., 2017). Compared to other studies that have been focusing on the impact of clinical state or age on microbiome data from infant to adulthood (Cox et al., 2010; Carmody et al., 2018), we focused on the use of volatility and machine learning to establish the important factors that are associated with the decline in lung function during the period of the life when a fast decline in lung function and an increase of the CF disease’s burden are observed (Szczesniak et al., 2017). Our data show that the temporal dynamics of the patient’s lung microbiome in a period of 3–5 years are linked with the individual microbiome composition and the dominance by Proteobacteria as well as the decline in the lung function. Patients with a diverse microbiome showed a high variability of the microbiome over time while patients carrying a monospecific and less diverse microbiome showed high stability that was associated with decrease in lung function. These results agree with a study of a cohort of adults showing a relatively stable microbiome dominated by Proteobacteria while patients with a more diverse microbiome had also a more variable microbiome composition (Whelan et al., 2017). Furthermore, our group-wise analysis comparing patients with lung function decline to stable patients showed that a milder disease progression in CF is linked to a volatile, dynamic, and diverse microbiome in the lower airways (**Figures 2**, **3**). Using a new analysis of the volatility of the lung microbiome, the necessity of multiple sampling becomes very obvious, since single data from just one time-point might be misleading (e.g., see the large variation in the volatility analysis) (**Figures 2**, **3**). Our findings highlight that analysis of single time-points holds the problem of high variance. Therefore, considerable changes in the lung microbiome can only be studied by longitudinal means combined with a volatility analysis.

High variability might indicate a cycle of bacterial immigration and removal due to a functional lung clearance system (Dickson et al., 2015). A stable microbiome in contrast is more probably linked to a pathogen-infected lung with local bacterial replication and worse outcome of the disease. In most of the patients presenting a higher diversity and higher temporal variability, the decline in lung function is reduced compare to patients with a high dominance of Proteobacteria. Yet, the question of which factors trigger the transition from an unstable but diverse microbiome to a microbiome dominated by chronic infection with a high abundant pathogen remains open. We propose to use the observed difference in the phylum abundances between the two patient groups identified here as a hint to an answer. Proteobacteria were the most responsible phylum for a chronically infected lung. Moreover, the phyla Bacteroidetes and Firmicutes were decreased and imbalanced for the “decliner.” Other studies have also reported associations between the reduction of *Prevotella* or in general anaerobic bacteria and the worsening of the lung function (Zemanick et al., 2013; O'Neill et al., 2015). Our data also demonstrate that the microbiome composition at a single time-point alone does not explain the classification of the patients in “stable” and “decliner” (**Figure 1**). The individual course and development are important, which can be analyzed in a longitudinal study. To our knowledge, this is the first analysis of the CF lung microbiome using the volatility analysis. The advantage of this approach is the use of machine learning (Random Forest) to highlight how microbial communities change over time. This method is useful to identify the influence of relative abundance and alpha-diversity associated not only with the overall temporal evolution but also with specific time points across individual courses. In our study, we run the model to find predictors of stability and decline in the lung function. The results emphasize the urgency to monitor the microbiome over time with a proper method. It becomes obvious that a single time-point analysis or comparison between two time-points can only give a snapshot and may lead to false impressions since the two curves in the volatility plot usually overlap strongly (**Figure 2**). Only the multiple and longitudinal analyses identify the average development and highlight the differences between the patient groups.

The role of Bacteroidetes and Firmicutes in a microbial community is a highly discussed topic in microbiome research (Fabbrizzi et al., 2019). A higher amount of short-chain fatty acids like acetate and butyrate were reported in individuals with increased Firmicutes abundance in the gut (Turnbaugh et al., 2006). Butyrate was shown in animal models to decrease oxidative stress in the gut and inhibit inflammation (Hamer et al., 2008). The lower abundance of Firmicutes in the “decliner” leads to the speculation that the level of butyrate could as well be decreased in this patient group. Assuming the butyrate amount in the lung also affects oxidative stress and inflammation, as it does in the gut microbiome; this could be a functional factor explaining our findings. Further analysis of metabolome products is necessary.

One of the main Proteobacteria found in our cohort was *Pseudomonas aeruginosa*. The lungs of four patients in this study cohort were chronically infected with *Pseudomonas*. Those patients showed a higher decrease in lung function as well as a microbiome which was more dominated by one ASV and thus less diverse but more stable. Chronic infections with the pathogen *Pseudomonas aeruginosa* were previously associated with lower diversity, higher dominance, increase in bacterial biomass of the lung microbiome (Boutin et al., 2017), and lower FEV_1_ values (Zemanick et al., 2013). In this longitudinal study, we confirm these observations partially, although there were also three patients with a moderate decrease of FEV_1_%pred per year and one patient with the highest worsening of the lung function without *Pseudomonas* infection. The microbiome of the latter patient revealed the occurrence of genera like *Aggregatibacter*, *Escherichia/Shigella*, *Eikenella*, *Bordetella*, *Stenotrophomonas*, and *Actinobacillus*, which are not characteristic for a healthy lung microbiome, and *Staphylococcus* which was already reported as a pathogen in CF (Akil and Muhlebach, 2018). Taken together, the infection with *Pseudomonas* correlates with the clinical data from the patients, but it cannot be the only reason for the decline in lung function of the CF patients.

Environmental factors are also affecting the lung function of CF patients by triggering exacerbation. I.v. antibiotic treatment was reported to increase lung function after an acute pulmonary exacerbation temporarily (Carmody et al., 2018). The higher frequency of antibiotic usage was linked to a worsening of lung function in the analyzed period in our study. However, this is a typical causality-consequence paradox and could be just a confounding factor because antibiotics have been the major improvement to treat pulmonary exacerbations in patients with CF. Whether the more frequent antibiotic therapies or the high frequency of clinical worsening led to a higher decline of FEV1%pred cannot be answered yet. Most likely, it is an interplay between both and other factors.

This study must be interpreted considering some limitations, which need to be addressed in future studies, like the relatively small cohort. Unfortunately, we also did not observe patients who started with a healthy-like microbiome and ended with a dominated microbiome to analyze the cause/consequence relationship between Proteobacteria and lung decline. The longitudinal analysis requires a repeated long-term sampling of the same patients; thus, we have a high number of samples with a low number of patients with the use of 16S rRNA gene-targeted sequencing. Further, we were limited in the depth of taxonomic analysis and were not capable to report virus or fungus abundances in the microbiome as well as information about existing genes, expression patterns, and biochemical pathways. Furthermore, we used spontaneous sputum expectoration as a proxy for the lower-airway state of health, limiting this study to patients who naturally expectorate sputum and therefore to patients with more severe lung diseases.

In summary, our data demonstrate that adolescents with CF showed an individual course of their lung microbiome over a 3–5-year period. The decrease in the variability of the microbiome was associated with the worsening of lung function mostly linked to a decline in the lung clearance and accumulation of PRPs. Furthermore, our data show that decliners present a disturbed microbiome dominated by a single species and a higher prevalence of i.v. antibiotic usage. While the benefit of antibiotics is undeniable, this makes one think about antibiotic stewardship and especially a shift toward more targeted therapy and alternative strategies.

## Data Availability Statement

The datasets presented in this study can be found in online repositories. The names of the repository/repositories and accession number(s) can be found as follows: NCBI BioProject, PRJNA759072.

## Ethics Statement

The studies involving human participants were reviewed and approved by the ethics committee of the University of Heidelberg (S 370/2011). Written informed consent to participate in this study was provided by the participants’ legal guardian/next of kin.

## Author Contributions

MAM, AD, and SB contributed to the conception and design of the study. SG, MS, and OS organized the clinical database. MIM and SB performed the bioinformatic and statistical analysis. MIM and SB wrote the first draft of the manuscript. All authors contributed to manuscript revision, read, and approved the submitted version.

## Funding

This study was supported in part by the German Ministry for Education and Research (82DZL00401, 82DZL004A1, and 82DZL009B1 to MAM, AD, and SB) and the German Research Foundation (SFB-TR84 TP B08 to MAM). SG is a participant in the BIH-Charité Clinician Scientist Program funded by the Charité–Universitätsmedizin Berlin and the Berlin Institute of Health.

## Conflict of Interest

The authors declare that the research was conducted in the absence of any commercial or financial relationships that could be construed as a potential conflict of interest.

## Publisher’s Note

All claims expressed in this article are solely those of the authors and do not necessarily represent those of their affiliated organizations, or those of the publisher, the editors and the reviewers. Any product that may be evaluated in this article, or claim that may be made by its manufacturer, is not guaranteed or endorsed by the publisher.
