# Supplementary material for: A Volatile and Dynamic Longitudinal Microbiome Is Associated With Less Reduction in Lung Function in Adolescents With Cystic Fibrosis
Source: Front Cell Infect Microbiol. 2021 Dec 6;11:763121. doi: 10.3389/fcimb.2021.763121 (PMC8687143; doi:10.3389/fcimb.2021.763121)

**Adolescents with cystic fibrosis with a volatile and dynamic longitudinal microbiome show less reduction in lung function**

Marisa I. Metzger ^1,2^, Simon Y. Graeber ^2,3,4,5,6,7^, Mirjam Stahl ^2,3,4,5,6,7^, Olaf Sommerburg^2,4^, Marcus A. Mall ^2,3,4,5,6,7^, Alexander Dalpke^1,2,8^, Sébastien Boutin^1,2#^

*Online Data Supplement*

### Sample preparation

After reception, samples are stored at 4°C and two hundred µl aliquots of Sputum samples were pre-treated with 50 µl PMA^TM^ dye (Biotium Inc., Hayward USA) in the first 24 h after sampling. The samples were incubated for 5 minutes in the dark and subsequently exposed to light (650-Watt, 20 cm distance to the samples). After sample mixing at 100 rpm, they were incubated for 10 minutes on ice. Living cells were pelleted by 10 minutes centrifugation at 5000 x g and resuspended in 200 µL PBS. Cells were stored at -20 °C.

### Quantitative PCR.

2 µl of purified DNA sample was mixed with 7.5 µl 1X Sybr-green master-mix (Life technology, Darmstadt, Germany). 50 pmol of each primer (forward primer: 5’-TGG AGG ATG TGG TTT AAT TCG A-3’; reverse primer: 5’-TGC GGG ACT TAA CCC AAC A-3’) was added, and the sample was filled up to 15 µl volume with H_2_O. In the thermal cycler (7900HT Fast Real-time PCF system (Applied Biosystems, Foster City, USA)), the duplicated samples underwent a first denaturation step at 95°C for 20 sec, followed by 40 amplification cycles (3 sec at 95°C, 30 sec at 60 °C). After two final steps at 95°C (15 sec) and 60 °C (1 min), the melt curve followed. The cycle threshold of each sample compared with the Cycle threshold values of plasmid DNA standard which had been cloned in-house and quantified by spectrophotometry [E1], quantifies the number of 16S copies.

### Statistics

The *longitudinal* plugin for Qiime2 [E2] was used to perform metadata Volatility Analysis and Feature Volatility Analysis on ASV-level and Phylum-level tables together with clinical values (n_estimators = 1000, estimator: Random Forest Regression). A time-index (t_i_) was introduced, to describe the time range of this study monthly as rational numbers and to align all patients accordingly to their age. The t_i_ was used as the state column and grouping of the longitudinal analysis was based on the affiliation to the “Stable” or “Decliner” patients’ group. The Feature Volatility Analysis uses the structure of the data as a learning input for a machine learning algorithm and identifies the feature (ASV or Phyla) which are the most important ones in the prediction of different states (time points).

**Supplement Reference**

E1. Nonnenmacher C, Dalpke A, Mutters R, Heeg K (2004) **Quantitative detection of periodontopathogens by real-time PCR.** Journal of Microbiological Methods 59: 117–125. pmid:15325758

E2. Bokulich NA, Dillon MR, Zhang Y, Rideout JR, Bolyen E, Li H, Albert PS, Caporaso JG: **q2-longitudinal: Longitudinal and Paired-Sample Analyses of Microbiome Data.** *mSystems* 2018, **3:**e00219-00218.

**Supplementary Figure**

**Supplementary Figure 1:** The samples for each patient are aligned to the time index (t_i_). Genera, which occur in at least one sample from a patient with a relative abundance ≥ 5 % are colored. The remaining genera are named “Others” and colored in gray. The order of the patients is conditional to their decline or increase of the FEV_1_%pred value per year. The antibiotic therapies are shown as orange circles, triangles or scares, which indicates the reason for the i.v. antibiotic therapy, on the x-axis and are as well aligned to the time index.


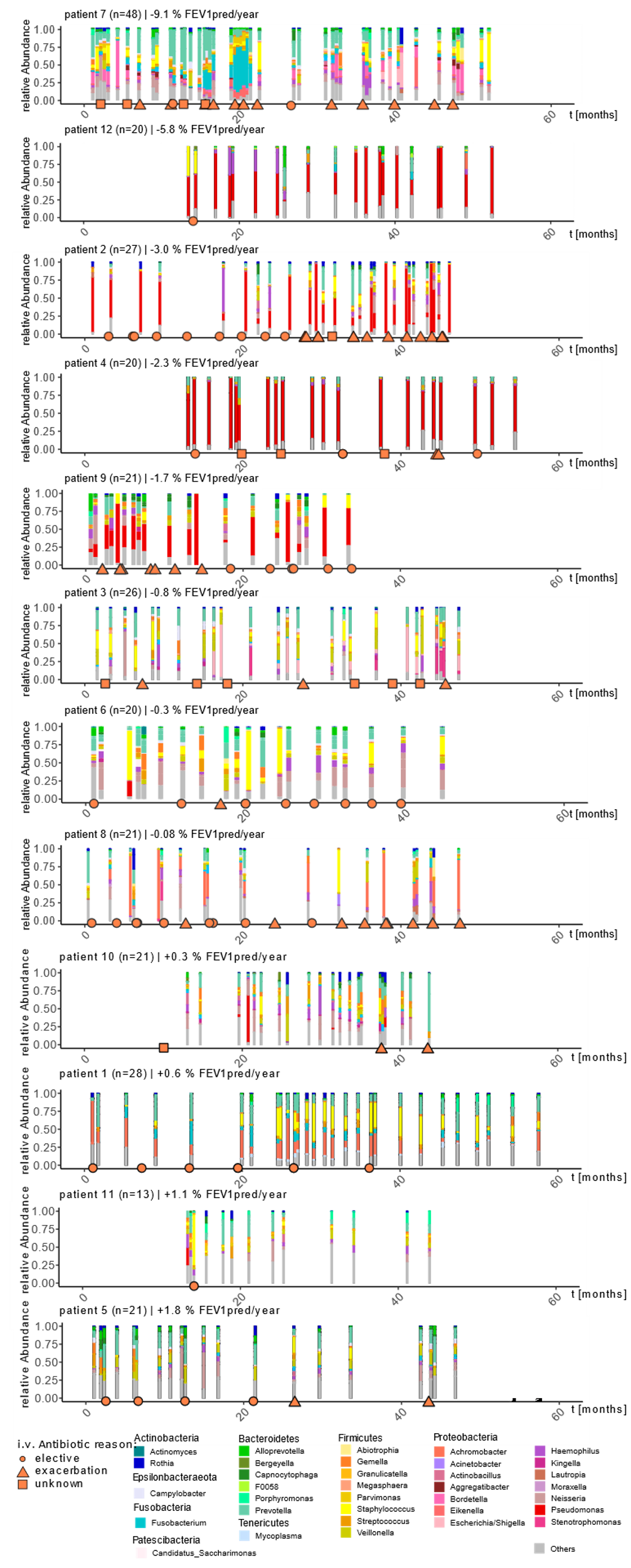


**Supplementary Figure 2:** The samples in the Pseudomonas cluster show a significant lower alpha and beta diversity compared to the samples from the other clusters. α-Diversity was calculated as the Shannon diversity and plotted for each sample according to their cluster affilitaion. β-Diversity was calculated as a matrix with the Morisita-Horn Calculation and the comparisons were grouped regarding to the cluster affiliation of the samples, thereby only comparisons within the same cluster (Pseudomonas vs. Commensals) have been kept. *** (p = 0.001), ** (p < 0.01), * (p < 0.05), NS. (not significant).

**Supplementary Figure 3:** The bacterial burden, measured by qPCR, is significantly higher in patient from the decliner group compared to the stable group.


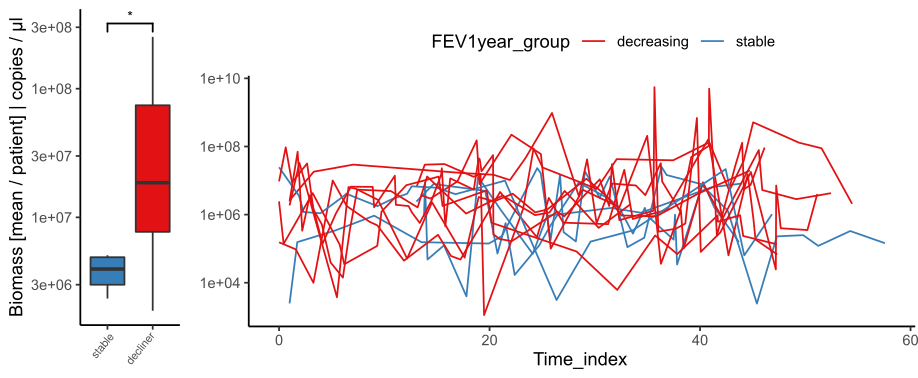

Supplement: Supplementary file 1 [file DataSheet_1.docx]
